# Inorganic Nanoparticle-Modified Poly(Phenylene Sulphide)/Carbon Fiber Laminates: Thermomechanical Behaviour

**DOI:** 10.3390/ma6083171

**Published:** 2013-07-26

**Authors:** Ana M. Díez-Pascual, Mohammed Naffakh

**Affiliations:** 1 Instituto de Ciencia y Tecnología de Polímeros (ICTP-CSIC), Juan de la Cierva 3, 28006 Madrid, Spain; 2 Universidad Politécnica de Madrid, Departamento de Ingeniería y Ciencia de los Materiales, Escuela Técnica Superior de Ingenieros Industriales, José Gutiérrez Abascal 2, 28006 Madrid, Spain; E-Mail: mohammed.naffakh@upm.es

**Keywords:** hybrid composites, IF-WS_2_ nanoparticles, poly(phenylene sulphide), porosity thermomechanical properties, synergistic effects

## Abstract

Carbon fiber (CF)-reinforced high-temperature thermoplastics such as poly(phenylene sulphide) (PPS) are widely used in structural composites for aerospace and automotive applications. The porosity of CF-reinforced polymers is a very important topic for practical applications since there is a direct correlation between void content and mechanical properties. In this study, inorganic fullerene-like tungsten disulphide (IF-WS_2_) lubricant nanoparticles were used to manufacture PPS/IF-WS_2_/CF laminates via melt-blending and hot-press processing, and the effect of IF-WS_2_ loading on the quality, thermal and mechanical behaviour of the hybrid composites was investigated. The addition of IF-WS_2_ improved fiber impregnation, resulting in lower degree of porosity and increased delamination resistance, compression and flexural properties; their reinforcement effect was greater at temperatures above the glass transition (T_g_). IF-WS_2_ contents higher than 0.5 wt % increased T_g_ and the heat deflection temperature while reduced the coefficient of thermal expansion. The multiscale laminates exhibited higher ignition point and notably reduced peak heat release rate compared to PPS/CF. The coexistence of micro- and nano-scale fillers resulted in synergistic effects that enhanced the stiffness, strength, thermal conductivity and flame retardancy of the matrix. The results presented herein demonstrate that the IF-WS_2_ are very promising nanofillers to improve the thermomechanical properties of conventional thermoplastic/CF composites.

## 1. Introduction

Fiber-reinforced thermosetting laminates have been widely used in aerospace, marine and automobile industries [1,2] during the past few decades replacing traditional materials such as steel and aluminum due to their good engineering properties including high specific strength, stiffness and fatigue endurance, high damping and low coefficient of thermal expansion combined with low density [3]. Recent interest is growing in the use of thermoplastics (TPs) to replace thermosets as matrices for laminate fabrication due to some advantages, namely higher toughness, improved corrosion, impact resistance and damage tolerance, shorter manufacturing cycles, much longer shelf life, no refrigeration storage required as well as reprocessing and recycling possibilities [4]. Carbon fiber (CF) or glass fiber (GF) reinforced high-temperature TPs such as poly(etheretherketone) (PEEK), polyetherimide (PEI) and poly(phenylene sulphide) (PPS) have been extensively used in the aeronautic sector due to their excellent mechanical and tribological properties, chemical and moisture resistance, heat stability and low flammability, smoke and toxicity performance [4]. In particular, PPS-based composite are resistant to aggressive media, exhibit good dimensional stability and property retention after exposure to severe temperature-humidity conditions, inherent flame retardancy, very low water absorption, anti-ageing and excellent friction properties. Further, they can attain a high degree of crystallinity and are cheaper than PEEK based laminates. However, to date, few studies dealing with the behaviour of carbon fabrics reinforced PPS laminates have been published [5,6,7,8,9,10,11,12,13,14,15], and these were focused on investigating the effect of temperature, hygrothermal aging and thermal cycling on their mechanical performance. It was found that at temperatures higher than the glass transition of these materials, the quality of the fiber-matrix interfacial adhesion significantly decreased, resulting in reduced mechanical properties particularly under off-axis loading conditions, and their behaviour became highly time-dependent due to the viscoelastic nature of the TP matrix [6,8]. Moreover, their mechanical performance was strongly influenced by the degree of crystallinity of the matrix in the laminates [16], since the crystalline phase tends to increase the stiffness and tensile strength while the amorphous phase is more effective in absorbing impact energy.

On the other hand, nanoparticle reinforced polymers are attracting a lot of attention due to their unique properties resulting from the nanoscale structures. The extremely high specific surface area of the nanoparticles enables the formation of a large interphase in the composite and strong nanofiller-matrix interactions. The addition of nanometric inorganic fillers to CF-reinforced polymers has been found to improve the wear resistance, fatigue life, toughness and damping characteristics [17,18]. It has been reported that there is a synergistic effect between the CFs and the nanoparticles on mechanical and tribological properties, since the enhancements attained are higher than the sum of the individual effects of each of the reinforcements. Thus, much effort is now focused on the development of multiscale (also named hybrid or hierarchical) composites, in which a nanoscale reinforcement is utilized together with traditional microscale fibers. Amongst the most promising types of nanoreinforcements are inorganic nanotubes (INT) and fullerene-like (IF) nanoparticles (e.g., layered metal dichalcogenides such as WS_2_ and MoS_2_) [19], which possess extraordinary properties such as very high stiffness and strength, and are strong enough to withstand uniform pressures up to 21 GPa [20]. Their outstanding mechanical properties can be attributed to their small size (typically in the range of 40–180 nm), quasi-spherical shape, closed-cage layered structure and chemical inertness. Moreover, they are cheaper than other organic nanofillers such as carbon nanotubes or nanofibers, and exhibit a lower tendency to form agglomerates, hence can be more homogenously dispersed within polymer matrices. Further, the IF nanoparticles exhibit excellent solid lubricant behaviour, and their efficiency as lubricant additives for improving the tribological properties of epoxy [21] and thermoplastic polymers [22,23] has been recently demonstrated.

In a previous work [24], IF-WS_2_ nanoparticles were used to fabricate hierarchical PPS/IF-WS_2_/CF composites, and the experimental results showed important improvements in the thermal stability, friction and wear properties compared to binary CF-reinforced PPS. The present study is aimed at investigating in detail the role of these nanoparticles on the porosity and mechanical performance of conventional PPS/CF laminates. Thus, the effect of IF-WS_2_ content on the interlaminar shear, flexural and compression properties, which are the most commonly used to provide design allowables for structural composite materials, is investigated at temperatures above and below the glass transition (T_g_). It is important to characterize the high-temperature behaviour of these laminates in order to find out whether they are suitable for use in transport applications, specifically in certain parts of the aircraft that demand a high service temperature (*i.e.*, ~120 °C for nacelles of the plane engines). Further, the influence of these nanoparticles on different thermomechanical properties such as coefficients of linear thermal expansion, heat deflection temperature and T_g_ as well as on the thermal behaviour of the laminates, including flammability and thermal conductivity, is also analyzed.

In the following section, a detailed discussion of the experiments performed is given. In Section 3, the materials, manufacturing of the laminates (stacking sequence, consolidation cycle, *etc*.), porosity measurements and characterization techniques are described. Finally, some conclusions are drawn.

## 2. Results and Discussion

### 2.1. Mechanical Behaviour

#### 2.1.1. Interlaminar Shear Tests

The interlaminar shear strength (ILSS) is one of the most important parameters in determining the ability of a composite material to resist delamination damage. The short-beam shear (SBS) test is a method frequently used to measure the apparent ILSS of fiber-reinforced composite laminates. Although it is not ideal to provide design allowables [25], it gives useful information about the strength of the fiber/matrix interfacial adhesion. Table 1 summarizes the degree of porosity and the short-beam shear strength (*σ_sbs_*) values for the reference PPS/CF and the hierarchical PPS/IF-WS_2_/CF laminates at 25 and 120 °C. *σ_sbs_* depends mainly on three factors [26]: resin tensile strength at failure; composite composition (nature of the constituents, architecture and concentration of the reinforcement); and level of porosity. The effect of voids is conditioned by their size. The larger voids acts as crack initiation sites under shear stress, whereas the increase in stress due to the reduction in net cross-section is the main cause of failure in composites with small distributed voids. There is a direct correlation between void content and interlaminar shear strength; it has been reported that the interlaminar shear decreases by 4%–8% per each 1% of voids [27]. Therefore, the strong rise in *σ_sbs_* observed in the presence of the nanoparticles (by up to 19% for the laminate with the highest IF-WS_2_ content in comparison to that of PPS/CF at 25 °C) should be related to the differences in IF-WS_2_ concentration, and cannot be solely justified by the variations in the degree of porosity. A similar trend is observed at 120 °C, albeit with higher increases, up to 32% at 2.0 wt % IF-WS_2_. Comparing the results obtained at both temperatures, it seems that the mechanical response of the laminates is the same from the elastic point of view, but the magnitude of *σ_sbs_* decreases at 120 °C due to the ductile behaviour of the PPS chains that is enhanced at T > T_g_. The plastic deformation of the matrix has a detrimental effect on the load transfer by shear between the CF plies; the plasticization dissipates part of the mechanical load applied, and consequently, the coupon is expected to fail mainly in tension and compression. Nevertheless, the nanoparticles can prevent the propagation of both interlaminar and intralaminar cracks, and hence reduce the deterioration of the ILLS with temperature. Thus, a 30% decrease in *σ_sbs_* is observed for the reference PPS/CF when the temperature is increased from 25 to 120 °C, while for the laminate with 2.0 wt % IF-WS_2_ the reduction is only 22%.

**Table 1:** Degree of porosity and short-beam shear strength *σ_sbs_* of PPS based laminates at 25 and 120 °C.

Laminate material	Porosity (%)	*σ_sbs_* (Mpa)	
		25 °C	120 °C
PPS/CF	2.0 ± 0.2	19.2 ± 1.1	13.3 ± 0.6
PPS/IF-WS_2_(0.1 wt %)/CF	1.8 ± 0.1	19.3 ± 1.5	13.3 ± 0.8
PPS/IF-WS_2_(0.5 wt %)/CF	1.4 ± 0.2	20.3 ± 0.8	15.4 ± 1.3
PPS/IF-WS_2_(1.0 wt %)/CF	0.9 ± 0.1	21.6 ± 1.7	16.7 ± 0.9
PPS/IF-WS_2_(2.0 wt %)/CF	0.6 ± 0.1	22.8 ± 1.2	17.5 ± 1.4

**Figure 1:**
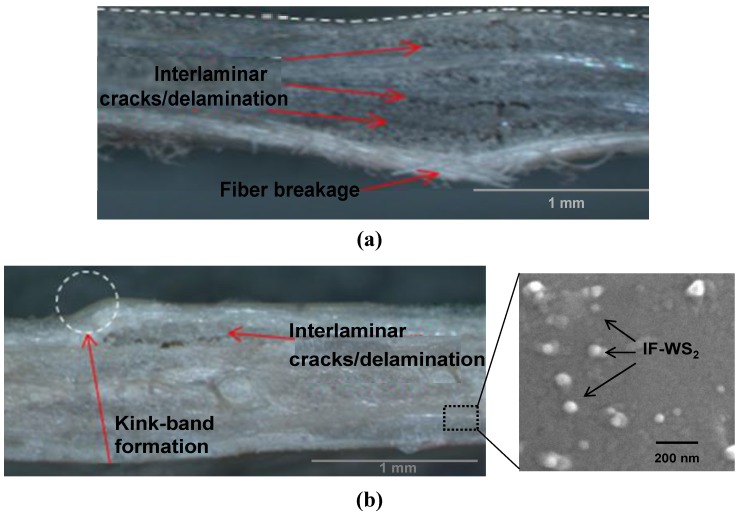
Through-thickness optical microscopy images of failed specimens after short-beam shear tests at 120 °C. (**a**) PPS/CF; and (**b**) PPS/IF-WS_2_ (1.0 wt %)/CF laminate. The right image in (**b**) is a SEM micrograph showing the IF-WS_2_ nanoparticles.

**Figure 2:**
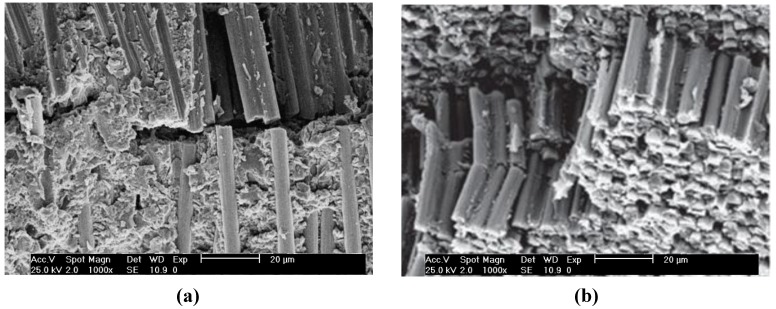
SEM observations after failure of SBS coupons at 120 °C. (**a**) PPS/CF; and (**b**) PPS/IF-WS_2_ (1.0 wt %)/CF laminate.

To obtain more information about the mechanisms responsible for the failure of the different laminates, the surfaces of failed coupons were examined by optical microscopy, and typical images of PPS/CF and PPS/IF-WS_2_ (1.0 wt %)/CF after SBS tests at 120 °C are shown in Figure 1. The image of the reference laminate (Figure 1a) shows several interlaminar cracks/delaminations predominantly following the neutral axis due to local debonding at the fiber-matrix interface, and these cracks propagate perpendicular to the load direction. Some fibers are found to be broken, suggesting that the lower and upper plies of the coupon failed primarily under tension and compression, respectively. In contrast, the image of the laminate with 1.0 wt % IF-WS_2_ (Figure 1b) reveals the presence of only a few interlaminar cracks, and the failure seems to be dominated by kink-band formation [28] at lower failure stresses. SEM micrographs of SBS coupons of the indicated laminates (Figure 2a,b, respectively) also confirm these findings. Kinking at 120 °C was observed for the laminate with the highest nanoparticle content, while those with IF-WS_2_ loading ≤0.5 wt % exhibited fiber breakage, as well as tension and compression failure in the outer plies, quite similar to the behaviour found for the reference composite. Clearly, as the nanoparticle concentration rises, the amount of interlaminar cracks is reduced and the material fails mainly in compression showing kink-band formation. An analogous trend was observed at 25 °C, where the number and size of the cracks progressively dropped with increasing IF-WS content. Optical microscopy images indicated that, in general, at T < T_g_, shear cracking and fiber/matrix debonding predominated, while at T > T_g_, the failure was dominated by kinking and CF fracture. Overall, it can be concluded that the role of the IF-WS_2_ at both temperatures is to act as barriers to crack propagation within the matrix region, thereby increasing delamination resistance by different mechanisms of interaction with the advancing cracks, such as localized inelastic matrix deformation.

#### 2.1.2. Compression Tests

**Figure 3:**
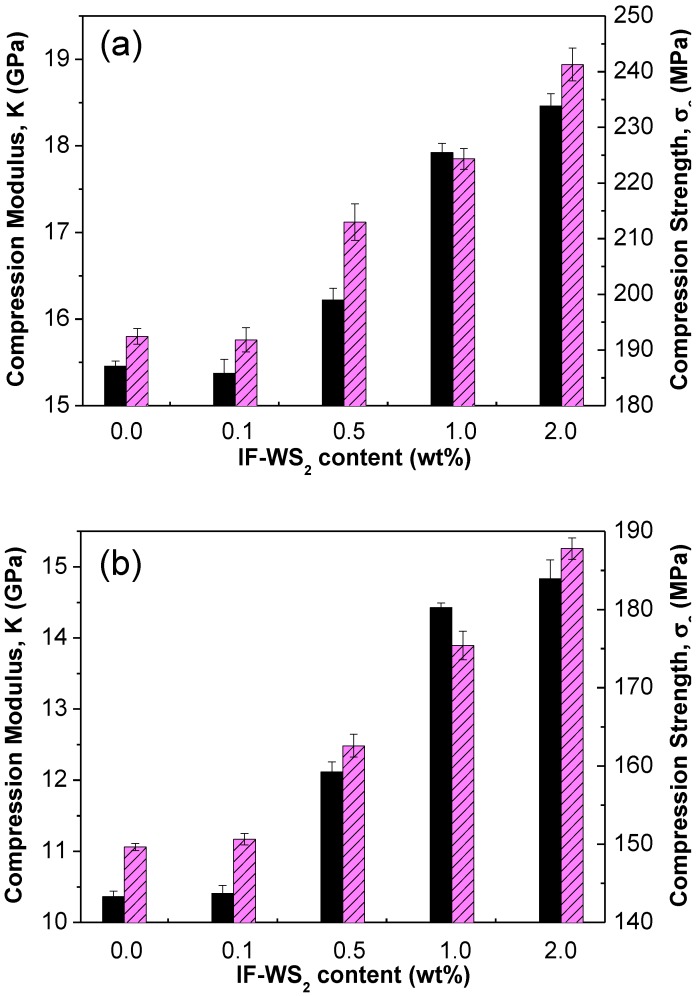
Effect of the IF-WS_2_ nanoparticles on the compression modulus K (solid bars) and strength σ_c_ (dashed bars) of PPS/CF laminates. (**a**) 25 °C; and (**b**) 120 °C.

In order to investigate the deformation of the multiscale laminates under compression and obtain information about their stiffness, a series of quasi-static experiments were carried out at 25 and 120 °C, and the results are displayed in Figure 3. At 25 °C, the reference PPS/CF shows a compression modulus (K) of around 15.4 GPa (Figure 3a); the addition of 0.1 wt % IF-WS_2_ results in a small decrease in K, while the incorporation of higher loadings gradually increases the modulus, by up to 20% at 2.0 wt % nanoparticle content. This behaviour should be related to the degree of crystallinity (X_c_) of the laminates (Table 2), since X_c_ of the sample with 0.1 wt % loading is lower than that of the reference, but increases for concentrations >0.5 wt % due to heterogeneous nucleation [29,30], and the crystalline regions are known to enhance the modulus of semicrystalline polymers [31]. Further, the increase in K at higher IF-WS_2_ contents is ascribed to the reduction in the degree of porosity, since the presence of voids also plays a key role in the compressive behaviour of the material. Nevertheless, the most important factor influencing K should be the conventional reinforcement effect. The nanoparticles confine the segmental movements of the PPS chains, resulting in higher modulus values. With regard to the compression strength σ_c_, the trends observed are qualitatively similar to those described for the modulus, and the largest increment (~25%) is found for the laminate with 2.0 wt % loading. At 120 °C (Figure 3b)—a temperature above the glass transition of the materials—there is a clear deterioration in properties, with an average reduction in K and σ_c_ of about 30% and 22%, respectively, caused by a decrease in the compression properties of the matrix due to the increased plasticization occurring at high temperatures. The laminate with 0.1 wt % loading displays similar K and σ_c_ values to the reference PPS/CF, indicating that the reinforcement effect at this temperature compensates for the decrease in crystallinity. At higher IF-WS_2_ concentrations, the increments in both parameters are larger than those observed at 25 °C, once again showing maxima at 2.0 wt % IF-WS_2_, corresponding to around 42% and 26% increases in K and σ_c_, respectively. The reinforcement effect of the nanoparticles is greater at T > T_g_ since they hinder the plastic deformation of the matrix, and their modulus is not expected to change with temperature. The results confirm that the matrix-dominated properties such as compression are enhanced by adding nanofillers, as previously reported for epoxy/CF composites incorporating graphite nanoplateles [32], and the extent of the improvement depends strongly on the loading, shape and state of dispersion of the nanoreinforcements, the degree of the filler-matrix interfacial adhesion as well as environmental factors such as temperature. The significant improvements in the mechanical properties of PPS/CF observed under compression should arise from the very homogenous nanoparticle dispersion attained through the melt-blending process [24], the quasi-spherical shape of the IF-WS_2_ that maximizes the contact area with the polymer, the minimization of internal porosity, an effective load transfer induced by strong interfacial adhesion coupled with a synergistic effect of both fillers on enhancing the matrix stiffness and strength. In fact, the addition of 2.0 wt % IF-WS_2_ to neat PPS only improved the matrix modulus and strength by ~10% and 12%, respectively, while the incorporation of the same amount of nanoparticles to PPS/CF increased them by 20% and 25%, thus corroborating the synergistic effect.

#### 2.1.3. Flexural Properties

**Figure 4:**
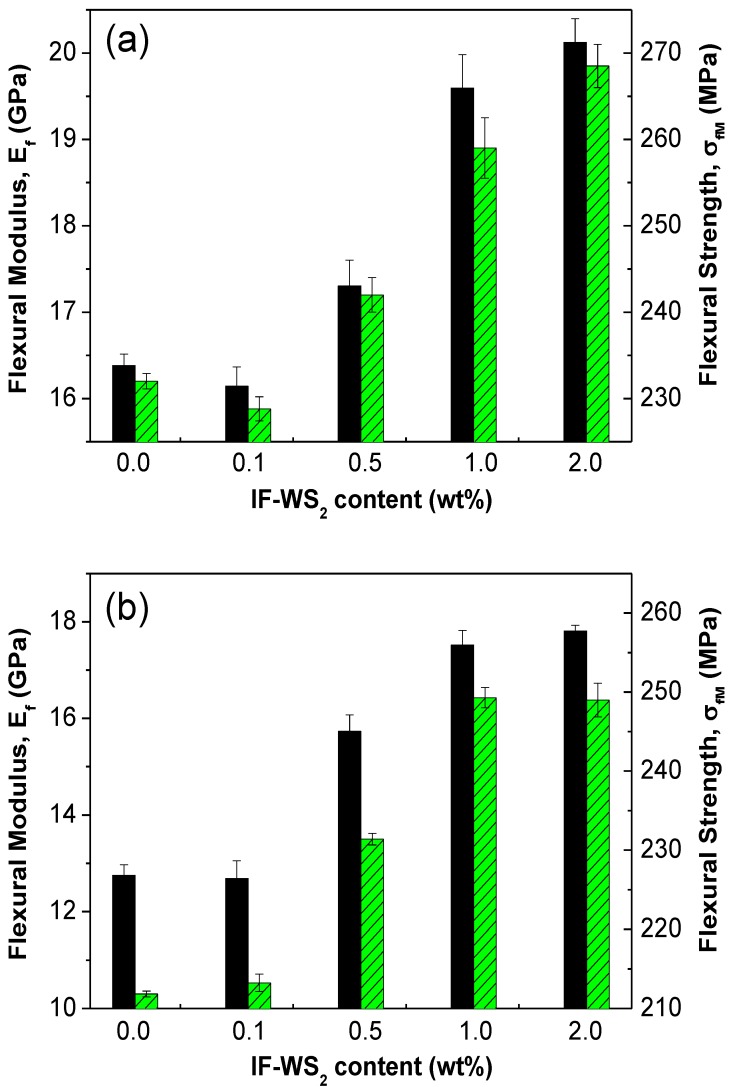
Flexural modulus *E_f_* (solid bars) and strength *σ_fM_* (dashed bars) for PPS/IF-WS_2_/CF laminates. (**a**) 25 °C; and (**b**) 120 °C.

The influence of the IF-WS_2_ nanoparticles on the flexural properties of PPS/CF at two different temperatures was also investigated and the results are presented in Figure 4. At 25 °C (Figure 4a), composites with very low IF-WS_2_ loading (0.1 wt %) exhibit slightly lower flexural modulus (*E_f_*) and strength (*σ_fM_*) than the reference PPS/CF, associated to the aforementioned decrease in the crystallinity of the matrix. In contrast, hybrid laminates with higher loadings show significant increments in both parameters, by up to 25% and 15% at 2.0 wt % IF-WS_2_, respectively. The remarkable improvements attained upon incorporation of these strong and stiff nanoparticles [33] are ascribed to their reinforcement effect in the *z*-direction, since the flexural properties are more matrix-dominated than fiber-dominated.

The results obtained from flexural tests performed at 120 °C (Figure 4b) indicate that the addition of 0.1 wt % loading hardly modifies neither *E_f_* or *σ_fM_* of PPS/CF, analogously to the behaviour described for the compression properties, while at higher concentrations the increments are again superior to those found at 25 °C (about 39% and 17% in modulus and strength, respectively, at 2.0 wt % IF-WS_2_). Once more, there is a reduction in stiffness and strength at T > T_g_, with average decreases of around 15% and 9%, respectively. Nevertheless, the reductions in flexural properties are less significant than those found in the compressive properties. This seems reasonable taking into account that matrix dependent properties such as compression are more affected by increasing temperature than fiber-dominated properties (*i.e.*, tension) [6], and that during the flexural tests, the outer layers of the laminates are mainly subjected to tensile and compressive stresses. This also may explain the fact that at high temperatures the nanoparticles exert more influence on the compressive behaviour (particularly on the strength) than on the flexural properties.

According to the results from the different mechanical tests, it is clear that IF-WS_2_-reinforced PPS/CF laminates are suitable for use in transport application at T > T_g_. Despite that there is a reduction in mechanical properties at 120 °C that oscillates between 30% and 9% depending on the type of test, the decrements are compensated or even overweighed by the reinforcing effect of the nanoparticles (see Table 1, Figure 3 and Figure 4), which is found to be more significant at higher temperatures. Therefore, these laminates have great potential to be used in high-temperature structural applications.

### 2.2. Thermomechanical Properties

#### 2.2.1. Glass Transition Temperature

The glass transition temperature (T_g_) of a hierarchical laminate typically differs from that of the neat resin because it is sensitive to matrix/reinforcement interfacial interactions both at the micro- and nano-scale. This characteristic temperature is indicative of the motion of the polymer chains; in an unfilled system, the chain segments are free from restraints. The addition of fillers decreases the free volume and generally restricts the mobility of the matrix chains, thereby increasing T_g_. Table 2 summarizes the T_g_ values of the different hybrid laminates obtained by thermomechanical analysis (TMA) measurements.

**Table 2:** Thermal conductivity (*λ*), degree of crystallinity (X_c_), heat deflection temperature (HDT), glass transition temperature (T_g_) and coefficient of thermal expansion (CTE) of PPS/IF-WS_2_/CF laminates.

Laminate material	HDT (°C)	X_c_ (°C) ^a^	T_g_ (°C)	CTE (10^−6^/°C)		*λ* (W/mK)	
				25 °C	120 °C	25 °C	120 °C
PPS/CF	253	28.2	98	47	115	0.93	1.09
PPS/IF-WS_2_(0.1 wt %)/CF	252	22.1	93	46	112	0.94	1.11
PPS/IF-WS_2_(0.5 wt %)/CF	259	25.6	96	43	106	0.96	1.15
PPS/IF-WS_2_(1.0 wt %)/CF	264	30.4	112	41	96	0.97	1.18
PPS/IF-WS_2_(2.0 wt %)/CF	270	34.5	118	38	79	0.99	1.23

^a^ data obtained from differential scanning calorimetry (DSC) analysis [24].

A strong dependence of T_g_ on the nanoparticle content was found. Laminates with IF-WS_2_ loadings ≤0.5 wt % showed a small downshift in this transition temperature compared to PPS/CF, whereas those with higher nanoparticle contents displayed an upshift, by up to 20 °C at 2.0 wt %. This unexpected behaviour may be related to the change from retardation to promotion in the crystallization rate of PPS with increasing IF-WS_2_ concentration, as revealed by DSC analysis [24]. Thus, low nanoparticle contents reduced the crystallization rate of PPS, resulting in the formation of a more amorphous phase, thereby leading to a slight decrease in T_g_, while at higher loadings they acted as heterogeneous nucleating sites for the matrix crystallization, raising its crystallinity. This combined with a larger matrix/IF-WS_2_ interfacial contact area provoked an effective immobilization of the polymer chains. Moreover, these nanoparticles possess solid lubricant character and reduce the viscosity of the matrix [23], which would allow additional mobility of polymer molecules. This effect could prevail over the restriction in mobility at low IF-WS_2_ concentrations and contribute to the unexpected decrease in T_g_. The raise in T_g_ at higher loadings could also be related to the increase in the rigid amorphous fraction of the nanocomposites with increasing content of IF-WS_2_, as previously reported for other nanocomposites reinforced with inorganic fillers [34].

On the other hand, there is a clear correlation between nanoparticle content (hence, material T_g_) and mechanical properties. Thus, for mechanical tests performed at 120 °C, PPS/CF is significantly above the T_g_, hence there is a highly ductile behaviour of the matrix, while PPS/IF-WS_2_ (2.0 wt %)/CF is very close to the T_g_, therefore is experiencing a transition from a hard and relatively brittle state to a soft and ductile state. Consequently, it presents considerably higher modulus and strength.

#### 2.2.2. Coefficient of Thermal Expansion (CTE)

The CTE is an important thermomechanical property used in the design of polymer composites for engineering applications. A low CTE is desirable to maintain the dimensional stability of the material. In fiber reinforced laminates, a low CTE in the direction perpendicular to the fibers can be obtained by dispersing hard nanofillers with low CTE inside the matrix. Inorganic nanoparticles are known to have low CTE [35], hence it is expected that the addition of IF-WS_2_ should have a notable effect on this property. Fibers play a dominant role in restricting the thermal expansion in the fiber direction, thus little influence of the nanoparticles would be in this direction. In contrast, matrix properties control the transverse direction, where the resin has less limitation to expand or shrink. Table 2 reports the CTE data at 25 and 120 °C measured across the thickness of the coupons using the TMA technique. In the glassy state, the CTE of the reference PPS/CF was found to be 47 × 10^−6^/°C, and this value decreased gradually upon addition of increasing IF-WS_2_ loadings, by up to 19% for PPS/IF-WS_2_ (2.0 wt %)/CF. Such noticeable reduction can be ascribed to the reduction in the degree of porosity and the increase in the rigidity of the matrix due to the presence of these nanoparticles that decrease the free volume of the polymer network and hinder the expansion of the PPS chain segments, restricting its thermally induced movements. This effective reduction also arises from the characteristics of the nanoparticles (*i.e.*, high modulus, spherical shape and low CTE), as well as from their very homogenous dispersion within the matrix that provides a high level of polymer constraint at very low concentration. As expected, the CTE values at 120 °C are more than double those measured at room temperature, and the trend observed is qualitatively similar for both test temperatures, albeit the CTE decrease for a given nanoparticle concentration is more pronounced at T > T_g_, being about 31% for the laminate with the highest IF-WS_2_ loading compared to that of PPS/CF. The stronger CTE reduction above T_g_ could be due to the more extreme differences in stiffness and thermal expansion between the polymer matrix and the nanofiller. Further, these nanoparticles possess a large specific surface area, hence would greatly absorb the heat transferred from the matrix, and in turn, significantly suppress the thermal expansion of the plastic polymer at T > T_g_.

#### 2.2.3. Heat Deflection Temperature (HDT)

HDT is the temperature at which a polymer sample deforms under a specified load, and is an important parameter when a material is being used for high-temperature structural applications. The presence of fibers has been demonstrated to significantly increase the HDT of polymers [36]. Also, the incorporation of hard nanofillers such as clay [37] or carbon nanotubes [38] into thermoplastic matrices has also been reported to enhance the HDT; thus, the HDT of laminate composites is expected to rise upon addition of nanoscale inorganic particles such as IF-WS_2_. The HDT data for the laminates are presented in Table 2. It can be observed that the incorporation of 0.1 wt % IF-WS_2_ leads to a slight decrease in this parameter, but it increases at higher loadings, up to 17 °C at 2.0 wt % nanoparticle concentrations. This improvement should stem from the rise in stiffness and strength as well as from the good dispersion of the nanoparticles that provide effective reinforcement to the PPS matrix. In general, there are three routes to increase the HDT of a polymer: increasing T_g_, raising the crystallinity and reinforcing. The small reduction in HDT upon addition of very low nanoparticle contents should be related to the decrease in crystallinity and T_g_ observed for this laminate (Table 2), as discussed previously, combined with the slight drop in stiffness, as revealed by compression and flexural data. The laminate with 0.5 wt % IF-WS_2_ also displays slightly lower X_c_ and T_g_ than the reference PPS/CF. However, the HDT is about 6 °C higher, indicating that the reinforcement effect overweighs the negative influence of the other two parameters. Regarding laminates with higher IF-WS_2_ loadings, the three factors influencing HDT are found to increase leading to a moderate rise in this property. The results demonstrate that multiscale PPS/IF-WS_2_/CF laminates are very suitable for high-temperature applications.

### 2.3. Thermal Behaviour

#### 2.3.1. Thermal Conductivity

**Figure 5:**
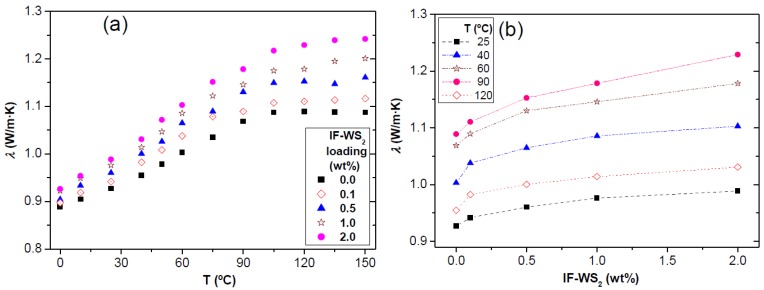
(**a**) Comparison of the thermal conductivity (*λ*) as a function of temperature for PPS/IF-WS_2_/CF laminates; and (**b**) *λ* *vs.* IF-WS_2_ nanoparticle loading for different temperatures.

The thermal conductivity (*λ*) of polymers can be enhanced by the incorporation of thermally conductive fillers such as graphite, carbon nanotubes, CFs, ceramic or metallic particles, to be used in certain applications that require effective dissipation of accumulated heat (*i.e.*, connectors, thermal interface materials for microelectronics, thermal management materials for spacecraft and underground oil-drilling applications) [39]. It depends on several factors, including the filler purity, size, aspect ratio, concentration and state of dispersion, the nature, molecular weight and degree of crystallinity of the polymer as well as the porosity of the material. *λ* of semicrystalline thermoplastics such as PPS has been found to rise with crystallinity [40], and generally increases with temperature up to T_g_, while it decreases slightly above T_g_. The heat transport occurs through elastic vibrations of the lattice (phonons), and due to the phonon scattering at the interface between the amorphous and crystalline phases, the prediction of *λ* *vs*. temperature presents a high degree of complexity. The temperature dependence of *λ* for the manufactured laminates is compared in Figure 5a. In the glassy state, the value of *λ* for all the samples rises almost linearly with temperature due to the larger mean free path and less phonon-phonon scattering. The presence of the nanofillers reduces the degree of porosity and enhances the phonon propagation length, leading to higher *λ* values. The average slope in this region rises gradually upon increasing nanoparticle concentration, from 2.05 × 10^−3^ W m^−1^ K^−2^ for PPS/CF to 2.91 × 10^−3^ W m^−1^ K^−2^ for the laminate with 2.0 wt % IF-WS_2_. Taking into account that *λ* of these nanoparticles remains almost constant within the temperature range studied [33] while that of the CFs shows a pronounced increase due to a significant increment in the phonon population of these carbon materials [41], the observed rise in *dλ*/*d*T points towards the existence of a synergistic effect of both fillers on improving *λ*. In the rubbery state, the slope of *λ* *vs.* T shows a significant reduction for all the samples, likely to be caused by a modification of the phonon modes and the corresponding change in thermal transport. In particular, the thermal conductivity hardly changes with temperature for PPS/CF and the hybrid laminates with low nanoparticle loadings, while it increases slightly for those with IF-WS_2_ contents ≥1.0 wt %; this behaviour is probably related to the differences in the degree of crystallinity of the laminates.

Figure 5b plots the change in *λ* *vs.* IF-WS_2_ loading for various selected temperatures. A clear increase in thermal conductivity is observed upon addition of these nanoparticles both above and below T_g_, which should arise from their higher *λ* compared to that of PPS [42] combined with a very homogenous dispersion and a strong interfacial adhesion with the matrix chains that promotes the conduction of phonons at the interface and minimizes the matrix coupling losses. It should be noted that their effect is more pronounced at T > T_g_; thus, the maximum *λ* increment at 120 °C (~15%) is about double that found at 25 °C. However, the improvements attained at 25 °C are relatively low compared to those found in binary PPS/IF-WS_2_ nanocomposites (by up to ~45% at 2.0 wt % IF-WS_2_ [42], probably due to the very high *λ* of the CFs [43] that masks the effect of the nanofillers. The laminates contain only 0.1–2.0 wt % IF-WS_2_ but 59 wt % of CF, hence the fibers play a dominating role in the thermal conductivity properties and consequently, only subtle increases in *λ* of the laminates are detected. Overall, the results confirm that the simultaneous incorporation of both micro- and nano-scale fillers into PPS is an effective way for improving the thermal conductivity of the resulting hybrid composites.

For multiscale fiber-reinforced laminates, the thermal conductivity can be predicted by two phase modelling. Firstly, *λ*_c_ of PPS/IF-WS_2_ at 25 °C can be calculated following the rule of mixtures:

*λ*_c_ = *λ*_m_·V_m_ + *λ*_n_·V_n_ (1)

where *λ*_c_, *λ*_m_ and *λ*_n_ are the thermal conductivities of the composite, PPS matrix [42] and IF-WS_2_ nanoparticles [33], respectively; and V_m_ and V_n_ are the matrix and nanoparticle volume fractions. Dispersion of the IF-WS_2_ into the polymer results in a new matrix phase, and the fibers can be regarded as another continuous phase. A modified equation of the model derived by Thornburgh and Pears [44] to express the transverse thermal conductivity of fiber-reinforced composites can be subsequently used to calculate *λ*_H_ of the hybrids [43]:

*λ*_H_ = V_m_·*λ*_m_·(1 – V_f_) + [(A·*λ*_m_·*λ*_f_)/(V_f_·*λ*_m_ + V_m_·V_f_·*λ*_f_)] (2)

being A = V_f_·(1 + V_m_), where *λ*_f_ is the thermal conductivity of the CF in the transverse direction, taken as 10 W m^−1^ K^−1^ at 25 °C [43]; V_f_ is the fiber volume fraction and the rest of parameters were described previously. The predicted values for PPS/IF-WS_2_/CF laminates were in the range 14%–18% higher than the experimental data, which can be explained considering that the model provides an upper bound for the thermal conductivity since assumes perfect nanoparticle dispersion within the polymer, perfect contact between particles in a fully percolating network, ignores the influence of voids, and makes no allowance for direct fiber-to-fiber contact. Another reason that expected *λ* enhancements were not attained is the thermal interface resistance or Kapitza resistance that represents a heat flow barrier associated with the differences in the phonon spectra between the matrix and filler phases and weak contact at the interface, both of which result in phonon backscattering. On the other hand, the model does not consider synergistic effects taking place due to the presence of both fillers.

#### 2.3.2. Flammability

**Figure 6:**
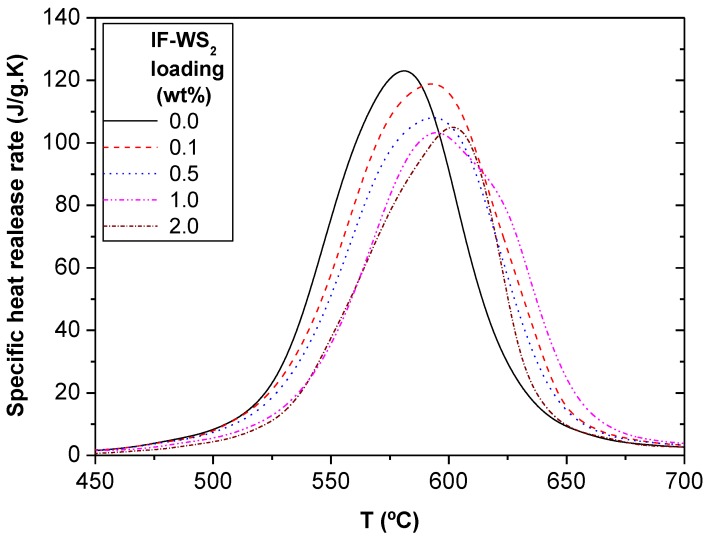
Heat release curves obtained from pyrolysis combustion flow calorimetry results for PPS/IF-WS_2_/CF laminates.

Recently, conventional thermally durable materials such as metals are being replaced with heat resistant high-performance polymer composites in applications where burn-through resistance and structural integrity after exposure to fire are required. The heat release rate (HRR) is the most important variable in fire hazard [45], particularly when dealing with composites used in enclosed spaces such as automobiles, ships or aircrafts. Therefore, it is interesting to assess the flammability behavior of PPS/CF and the hierarchical laminates. Their heat release curves obtained from pyrolysis combustion flow calorimetry are displayed in Figure 6, and the results are summarized in Table 3. As expected, the reference PPS/CF begins releasing heat prior to the hybrid laminates, since these nanoparticles are reported to increase the thermal stability of PPS matrix [42]. The addition of IF-WS_2_ leads to a progressive drop in the average peak HRR, from 122 W/g for the binary laminate to 101 W/g (a 17% reduction) at 1.0 wt % loading. Note that these inert nanoparticles do not decrease the peak HRR uniformly. A more rapid pyrolysis rate, hence release of flammable material, was found for the laminate with the highest IF-WS_2_ loading. The onset temperature at which begins the release of heat corresponds to the incipient ignition temperature (flashpoint) of PPS, while the temperature at peak HRR corresponds to the continuous ignition (steady burning) temperature [46]. Both temperatures increased gradually with the nanoparticle loading, with maximum increments of 19 and 23 °C, respectively, at 2.0 wt % IF-WS_2_. These improvements are most likely related to the lower degree of porosity and enhanced thermal conductivity of the hybrid laminates compared to that of PPS/CF, as discussed previously. The nanoparticles help to dissipate the heat quickly through the bulk of the laminate, which means that it takes longer for the surface temperature of the sample to reach the ignition point and the peak HRR. Another plausible explanation for the observed improvements could be that the IF-WS_2_ act as a mass transport barrier that hinder the escape of volatile products generated during the decomposition process and also prevent oxygen from reaching the matrix. Interestingly, the differences in the char yields between the reference PPS/CF and the multiscale composites correspond fairly well with the IF-WS_2_ concentration of the laminates. Given the low percentage of nanoparticles used in this work, the enhancements in flame retardancy attained point towards the existence of a synergistic effect of both fillers on increasing the polymer resistance to fire. The coexistence of CFs and IF-WS_2_ in the laminates results in a more effective confined geometry that increases the barrier resistance to the evolution of flammable volatiles. Similar synergistic behaviour has been reported for different polymer/clay/carbon nanotube hybrids [47,48].

**Table 3:** Pyrolysis combustion flow calorimetry data for PPS/IF-WS_2_/CF laminates.

Laminate material	T_onset_ (°C)	T_peak_ (°C)	Peak HRR (W/g)	Char yield (%)
PPS/CF	538	580	122	75
PPS/IF-WS_2_(0.1 wt %)/CF	545	591	118	76
PPS/IF-WS_2_(0.5 wt %)/CF	550	594	107	76
PPS/IF-WS_2_(1.0 wt %)/CF	556	595	101	77
PPS/IF-WS_2_(2.0 wt %)/CF	557	603	104	78

## 3. Experimental Section

### 3.1. Materials

**Figure 7:**
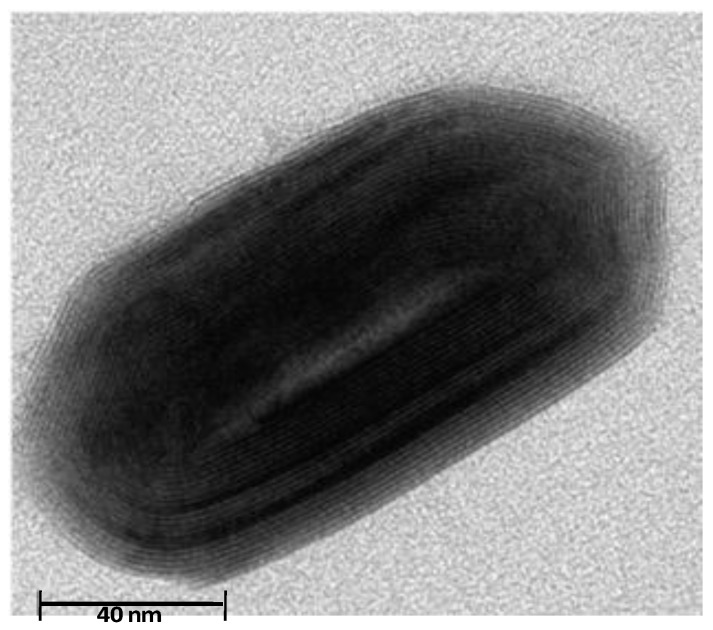
TEM micrograph of a hollow ellipsoidal IF-WS_2_ nanoparticle.

PPS (Fortron 02054P4, d_25 °C_ = 1.35 g/cm^3^, T_g_ = 90 °C, T_m_ = 280 °C) was kindly supplied by Ticona in pellet form. The polymer was dried at 100 °C for 14 h and stored in a dry environment before blending. Inorganic fullerene-like tungsten disulphide (IF-WS_2_) nanoparticles (NanoLub™, d_25 °C_ ~ 7.5 g/cm^3^) were provided by Nanomaterials (Yavney, Israel). They are closed-cage hollow multilayered polyhedral nanoparticles with an apparent shape ranging from spheres to ellipsoids (Figure 7). The particle aspect ratio ranges between 1 (spheres) and 2.3, with a mean value of 1.4 and standard deviation of 0.3. The most characteristic particle has a near ellipsoidal shape with diameter in the range of 40–150 nm (mean value of 80 nm). They have an onion-like structure and are composed of concentric WS_2_ layers evenly spaced by 6.18 Å. Its hollow nature is reflected by the contrast difference in the core, and the dimension of the hollow void in the center is about half the overall diameter of the nanoparticles. Standard modulus carbon fiber fabrics 5-harness satin (fabric reference G0926, areal weight = 370 ± 14 g/m^2^, average fiber diameter = 7 μm, fiber density = 1.77 g/cm^3^) was provided by Hexcell Reinforcements (Les Avenieres, France).

### 3.2. Manufacturing of PPS/IF-WS_2_/CF Laminates

The multiscale laminates were prepared via melt-blending and hot-press processing, techniques that are simple, environmentally friendly and easy to scale at an industrial level. Firstly, the polymer was physically mixed with different concentrations of IF-WS_2_ (0.1, 0.5. 1.0 and 2.0 wt % loading). Subsequently, each mixture was dispersed in 50 mL of ethanol and sonicated in an ultrasonic bath for 30 min. The dispersions were then dried under vacuum (70 mbar) at 50 °C until total evaporation of the solvent. The melt-blending of the solid dispersions was carried out using a Haake Rheocord 90 extruder operating at 320 ± 5 °C, with a rotor speed of 150 rpm and mixing times of 20 min. The resulting PPS/IF-WS_2_ extrudate was used to fabricate films with a thickness of ~0.2 mm in a hot-press at the same temperature under high pressure.

**Figure 8:**
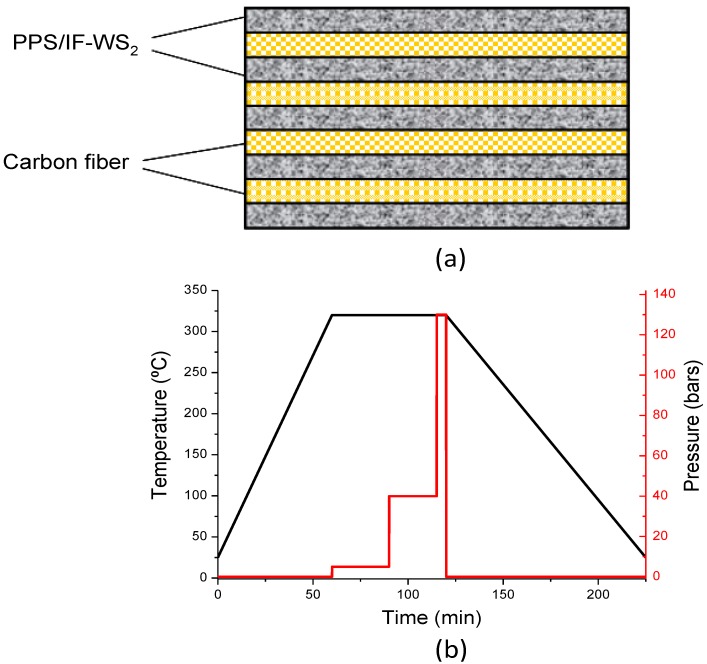
(**a**) Laminate stacking sequence; and (**b**) consolidation cycle used for the fabrication of the laminates.

The laminates were fabricated by hot-pressing at 320 °C, 4 plies of CF fabric alternatively placed between 5 PPS/IF-WS_2_ films, using successive pressure dwell steps of 5, 40 and 130 bars for periods of 30, 25 and 5 min, respectively. The heating rate to the dwell temperature was about 5 °C/min, and the cooling to room temperature was carried out slowly at a rate ≤3 °C/min. The laminate stacking sequence and the consolidation process cycle are displayed in Figure 8. The pressure steps were optimized to improve fiber impregnation. The resulting laminates had a nominal thickness of 1 ± 0.1 mm, a resin content of 35 ± 2 wt % and an average density of 1.62 ± 0.03 g/cm^3^.

### 3.3. Assessment of Density and Porosity

The density of laminate specimens (~10 × 10 mm^2^) was measured using a specific gravity determination kit equipped with an electronic balance (readability of 0.001 mg) at 20 °C. The values were calculated based on the Archimedes’ principle according to the equation:

*ρ_c_* = [*w_a_/w_a_ – w_w_*] × *ρ_o_* (3)

where *ρ_c_* is the density of the specimen; *w_a_* and *w_w_* the weight of the specimen in air and water, respectively; and *ρ_o_* the density of the water at that temperature. The theoretical density of the laminates was calculated using the equation:

*ρ_T_* = 100/[(*w_m_*/*ρ_m_*) + (*w_f_*/*ρ_f_*)] (4)

being *w_m_* and *ρ_m_* the weight percentage and density of the matrix; *w_f_* and *ρ_f_* the weight percentage and density of the reinforcement, respectively. The areal density of the CF was utilized to estimate *w_f_* and *w_m_*. The porosity was determined as difference between the experimental and the theoretical density:

Porosity (%) = [(*ρ_T_* – *ρ_c_*) / *ρ_T_*] × 100 (5)

The average porosity of all the samples is below 2.5% (Table 1); hence, they are well-suitable for structural applications. Interestingly, a significant reduction in porosity is observed upon increasing nanoparticle loading, by up to ~70% at 2.0 wt % IF-WS_2_. Taking into account that all the laminates were manufactured by the same processing and possess similar thickness and resin/fiber content, the differences in void content should be related to the presence of the IF-WS_2_. Indeed, these lubricant nanoparticles decrease the viscosity of PPS matrix [23], thus facilitating the viscous flow of the polymer chains, and improving fiber impregnation. With increasing particle loading, the melt-viscosity progressively drops [23], and therefore, the porosity is minimized. This finding is highly interesting since the incorporation of small amounts of these cheap nanoparticles can improve the quality of fiber-reinforced high viscosity thermoplastics through minimizing the formation of internal pores.

**Figure 9:**
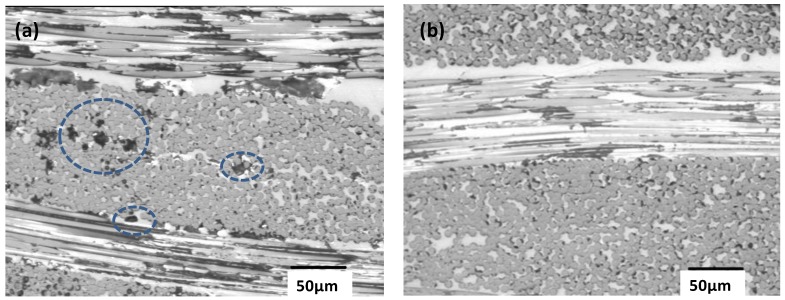
Optical microscopy images of (**a**) PPS/CF; and (**b**) PPS/IF-WS_2_ (1.0 wt %)/CF laminate. The dashed circles in (**a**) show macropores within the CFs.

To obtain further information about the material porosity, the laminates were examined by optical microscopy, and typical images of PPS/CF and PPS/IF-WS_2_ (1.0 wt %)/CF are shown in Figure 9. No cracks or delaminations were detected in the examined areas, hinting towards the good quality of the laminates. The carbon fiber fabric acts as a barrier to crack propagation [49]. Moreover, the nanoparticles also hinder crack growth. Therefore, the combination of both reinforcements results in improved resistance to crack growth and delamination. Two types of voids were found in PPS/CF as shown in Figure 9a: Spheroidal (predominant) and ellipsoidal formed between transverse fibre bundles. The pores are not homogenously distributed, they are primary present at the laminate center, and they do not have a uniform size distribution. Thus, macropores between 0.5 and 10 μm were observed in the images. In contrast, the laminate with 1.0 wt % IF-WS_2_ showed considerably lower level porosity (Figure 9b) with smaller voids, in agreement with the density measurements.

### 3.4. Characterization Techniques

Transmission electron microscopy (TEM) images were obtained with a Philips Tecnai 20 FEG (LaB_6_ filament) electron microscope fitted with an EDAX detector operating at 200 kV. Prior to characterization, ultra-thin sections of the composites were cut using a diamond knife and a Reichert Ultracut-S ultramicrotome equipped with a FCS cryo-device and placed on copper grids.

Quasi-static mechanical tests were carried out on a servo-hydraulic testing machine (type MTS 858, TestResources Inc., Shakopee, MN, USA) equipped with a temperature control system, using a crosshead speed of 1 mm/min and a load cell of 100 kN, in the temperature range between 25 and 120 °C, at 50% ± 5% RH. All the samples were conditioned for 24 h before the measurements. Five specimens were tested for each laminate, and the data reported correspond to the average value. The short beam strength (*σ_sbs_*) was calculated according to ASTM D2344 standard [50] as a function of the maximum load (*F*_max_), the width (*b*) and thickness (*h*) of each coupon:

(6) $$\sigma_{sbs}=0.75\frac{F_{\max}}{bh}$$

Compression tests were performed using a specific fixture to prevent buckling, as described in the Boeing-Modified ASTM D695 method [51]*.* To calculate the compression modulus (*K*), specimens without tabs were loaded to a strain of 0.3% using a strain gage to measure strain. Then, specimens tabbed at each end were tested to failure to determine the compressive strength (σ*_c_*). Flexural tests were carried out in accordance to ASTM D790 (three point loading, [52]). The flexural strength *σ_fM_* and flexural modulus *E_f_* were determined as:

(7) $$\sigma_{fM}=3\frac{F_{\max}L}{2bh^{2}},\;E_{f}=\frac{mL^{3}}{4bh^{3}}$$

where *L* is the support span and m the slope of the initial straight-line portion of the load deflection curve.

Fractography analysis was used to assess the failure mechanism of the PPS based laminates. Optical microscopy images at low magnification were obtained with a Nikon SMZ 1000 microscope (Melville, NY, USA) coupled to a digital camera to capture the images. The failure surfaces were examined at high magnification using a Philips XL30 scanning electron microscope (SEM) operating at 25 kV. Samples were coated with a ~5 nm Au/Pd overlayer to avoid charging during electron irradiation.

HDT was measured using a HDT/VICAT heat deflection tester according to ASTM D648 standard. Specimens were conditioned at 25 ± 2 °C and 50% ± 5% RH for 24 h prior to the measurements. The sample position was edgewise, test span 100 mm, the surface stress 1.8 MPa and the heating rate 2 °C/min.

The effective thermal conductivity (*λ*) of the laminates through thickness (in the transverse fiber direction) was determined with a KES-F7 Thermo Labo type II equipment (KATO Tech., Kyoto, Japan). The device is equipped with a temperature controlled hot plate, and is placed in a thermostatic chamber to be kept in a constant operating environment. The effect of the contact thermal resistance was removed by measuring the thermal conductivity of a reference material. *λ* was calculated using the equation *λ* = tW/(T_hot_ – T_cold_) A, where t is the sample thickness; W is the heat flow; T_hot_ and T_cold_ are the temperatures of the hot and cold plates, respectively; and A is the surface area of the hot plate (2.5 × 2.5 mm^2^). Five specimens for each laminate were tested and the average value is reported.

The CTE of the laminates was measured across the thickness using a Perkin-Elmer TMA 7 thermomechanical analyzer (Waltham, MA, USA). Samples of ~7 mm × 7 mm size were heated from 25 to 250 °C at rate of 2 °C/min under nitrogen atmosphere. T_g_ was identified as the temperature at which the slope of the TMA plot changed, and the CTE was determined both below and above T_g_.

The heat release rate (HRR) was obtained by pyrolysis combustion flow calorimetry on samples of ~10 mg. Experiments were carried out under nitrogen flow at a heating rate of 60 °C/min; the maximum pyrolysis temperature was 900 °C. The decomposition products were mixed with excess oxygen and completely oxidized at high temperature. The HRR was determined by oxygen consumption calorimetry. Prior to the measurements, samples were conditioned at 23 ± 2 °C and 50% ± 5% RH for 1 week. The materials were tested in triplicate to ensure good reproducibility.

## 4. Conclusions

The effect of IF-WS_2_ nanoparticles on the porosity, and thermal, mechanical and thermophysical properties of CF-reinforced PPS laminates has been studied. The mechanical performance of the hierarchical laminates was investigated at various stress states (compression, flexion and interlaminar shear) and two different temperatures, 25 and 120 °C. The nanoparticles increased the delamination resistance of PPS/CF at temperatures both above and below its glass transition (T_g_ = 98 °C), and the maximum increment (~32%) was attained at 120 °C and 2.0 wt % loading. Fractography analysis of failed coupons from SBS tests revealed a significant reduction in the amount and size of interlaminar cracks/delaminations with increasing nanoparticle content, related to the drop in the degree of porosity. Remarkable improvements in the compression and flexural properties were attained for IF-WS_2_ concentrations ≥1.0 wt %, arising from very homogenous nanofiller dispersion, an effective load transfer induced by strong polymer-nanoparticle interfacial interactions, combined with a lower level of internal porosity and a rise in the crystallinity of PPS. The influence of these nanoparticles on the matrix-dominated mechanical properties was found to be more pronounced at temperatures above T_g_. A strong dependence of the HDT and T_g_ on the nanofiller content was observed: the addition of IF-WS_2_ loadings ≤0.5 wt % led to a downshift in both parameters compared to those of the reference PPS/CF related to the decrease in crystallinity, while higher concentrations provoked a significant upshift due to the nucleating and reinforcing effect of these nanoparticles. Further, they hindered the thermally induced movements of the PPS chains, thus reducing the CTE both above and below T_g_. A noticeable rise in the transverse thermal conductivity was also detected. In the glassy state, the conductivity increased almost linearly with temperature, and the slope *dλ*/*d*T augmented steadily with the nanoparticle loading. In the rubbery state, this property hardly changed for the laminates with low nanofiller contents, while it increased slightly for those with concentrations ≥1.0 wt % attributed to differences in their degree of crystallinity. A combination of the rule of mixtures and the Thornburgh and Pears modified equation was used in hierarchy to predict the room temperature thermal conductivity of the multiscale laminates, and the differences between experimental and theoretical values were in the range of 14%–18%. These hybrid materials also exhibited superior fire performance: a remarkable drop in the average peak HRR was found combined with an increase in the ignition point and the steady burning temperature. The results suggest the existence of synergistic effects of both fillers on enhancing the stiffness, strength, thermal conductivity and flame retardancy of PPS matrix. The combination of CF and IF-WS_2_ nanoparticles is a simple, feasible and effective approach to improve the mechanical and thermal behavior of TP resins such as PPS to be used in a wide range of structural applications, primarily in the aeronautic and automotive sectors, both at temperatures above and below the T_g_.
